# COVID-19 Deaths: Are We Sure It Is Pneumonia? Please, Autopsy, Autopsy, Autopsy!

**DOI:** 10.3390/jcm9051259

**Published:** 2020-04-26

**Authors:** Cristoforo Pomara, Giovanni Li Volti, Francesco Cappello

**Affiliations:** 1 Department of Medical, Surgical Sciences and Advanced Technologies “G.F. Ingrassia”, University of Catania, 95100 Catania, Italy; 2 Head of the Medico-Legal Unit at University Hospital “Policlinico Vittorio, 95100 Catania, Italy; 3 Department of Biomedical and Biotechnological Sciences, University of Catania, 95100 Catania, Italy; 4 Department of Biomedicine, Neuroscience and Advanced Diagnostics, University of Palermo, 90100 Palermo, Italy; 5 Department of Biology, Temple University, Philadelphia, PA 19122-6078, USA

**Keywords:** COVID-19, infectious diseases, autopsy, diagnosis

## Abstract

The current outbreak of COVID-19 severe respiratory disease, which started in Wuhan, China, is an ongoing challenge, and a major threat to public health that requires surveillance, prompt diagnosis, and research efforts to understand this emergent pathogen and to develop an effective response. Due to the scientific community’s efforts, there is an increasing body of published studies describing the virus’ biology, its transmission and diagnosis, its clinical features, its radiological findings, and the development of candidate therapeutics and vaccines. Despite the decline in postmortem examination rate, autopsy remains the gold standard to determine why and how death happens. Defining the pathophysiology of death is not only limited to forensic considerations; it may also provide useful clinical and epidemiologic insights. Selective approaches to postmortem diagnosis, such as limited postmortem sampling over full autopsy, can also be useful in the control of disease outbreaks and provide valuable knowledge for managing appropriate control measures. In this scenario, we strongly recommend performing full autopsies on patients who died with suspected or confirmed COVID-19 infection, particularly in the presence of several comorbidities. Only by working with a complete set of histological samples obtained through autopsy can one ascertain the exact cause(s) of death, optimize clinical management, and assist clinicians in pointing out a timely and effective treatment to reduce mortality. Death can teach us not only about the disease, it might also help with its prevention and, above all, treatment.

## 1. Introduction

The coronavirus disease 2019 (COVID-19) pandemic is an ongoing challenge, a threat to global health that requires surveillance, prompt diagnosis, and research efforts to understand this emergent pathogen and to develop effective countermeasures.

## 2. Discussion

Due to scientific community’s efforts, there is an increasing body of published studies describing the COVID-19’s biology, its transmission and diagnosis, its clinical features, its radiological findings, and the development of candidate therapeutics and vaccines. Vice versa, very few autopsy-based data are yet available.

Are we sure that is it correct to treat COVID-19 as a severe pneumonia? Are we sure people are dying “with” and not “because of” COVID-19?

We have only one instrument in medicine to answer to these crucial questions: AUTOPSY, AUTOPSY, AUTOPSY!

Despite the decline in postmortem examination rate, it remains the gold standard to determine why and how death happens. Defining the pathophysiology of death is not only limited to forensic considerations, it may also provide useful clinical and epidemiologic insights [1,2].

Selective approaches to postmortem diagnosis, such as limited postmortem sampling over full autopsy, can also be useful in the control of disease outbreaks and to provide valuable knowledge for managing appropriate control measures [3,4]. Collecting cadavers’ samples or biological fluid swabs can be also useful in the control of epidemics, as shown during previous infectious disease outbreaks. During West Africa Ebola epidemic and for the Ebola Virus Disease (EVD) surveillance strategy, the RNA virus was isolated in body fluids days or months after the onset of the disease from any living or deceased individual who had, or had had, clinical symptoms compatible with EVD. Thanks to this procedure, it was possible to monitor the number of infected patients in order to recognize new sources of transmission and to control the epidemic phenomenon [5,6,7,8,9,10].

Many physicians are wondering these days if we are not facing a systemic pathology that affects the vessels of different anatomical districts, not only the lung but the heart, kidney, liver, intestine, brain, and even the skin. This hypothesis is supported by the fact that angiotensin-converting enzyme 2 (ACE2), and putatively also sialic acids, the supposed “doors” by which COVID19 enters into endothelial cells and pericytes, are almost ubiquitarian, and not only present in the endothelial cells of alveolar membrane [11,12].

How can we ever answer these colleagues without doing a good number of autopsies? Lack of data from autopsies might result in incomplete or even incorrect postmortem diagnosis during current COVID-19 outbreak. However, it is likely that the suddenness of the outbreak, the number of patients in hospitals, the shortage of healthcare personnel, and the high rate of transmissions [13] may significantly reduce the number of autopsies and sampling from cadavers. Clinical value of autopsy is also supported by several studies demonstrating that despite the introduction of more modern diagnostic techniques and of intensive and invasive monitoring, the number of missed major diagnoses has not essentially changed over the past 20 to 30 years; autopsies revealed ante mortem diagnostic errors or ante mortem unrecognized diagnoses in about 30% of cases [2,14].

We cannot, though, underestimate the importance of autopsy as a diagnostic tool to understand the underlying mechanisms behind death. In accordance with the World Health Organization, postmortem examination for deceased persons infected with COVID-19 should be consistent with those used for any autopsies of people who have died from an acute respiratory illness, following the recommended safety procedures. In this scenario, we strongly recommend performing full autopsies on patients who died with suspected or confirmed COVID-19 infection, particularly in the presence of several comorbidities. Only working a complete set of histological samples obtained through autopsy could help to ascertain the exact cause(s) of death, optimizing clinical management and assisting clinicians in identifying a timely and effective treatment to reduce mortality.

Moreover, the identification of the exact cause of death could be valuable in the near future, preventing legal and civil disputes for hospital personnel.

## 3. Conclusions

Reviving the practice of autopsy can provide useful information to match with clinical data in order to achieve a better understanding of the pathogenesis of this novel coronavirus disease. As a scientific community, we are called to face this global threat and to defeat it with all available tools needed, new and old, as the new and the old represent a proper union for continued progress in medicine.

Death can teach us not only about the disease, it might help with its prevention and, above all, treatment.
